# MiRNA-103/107 in Primary High-Grade Serous Ovarian Cancer and Its Clinical Significance

**DOI:** 10.3390/cancers12092680

**Published:** 2020-09-19

**Authors:** Milosz Wilczynski, Michal Kielbik, Daria Senderowska, Tomasz Krawczyk, Bozena Szymanska, Magdalena Klink, Jan Bieńkiewicz, Hanna Romanowicz, Filip Frühauf, Andrzej Malinowski

**Affiliations:** 1 Department of Operative Gynecology, Endoscopy and Gynecologic Oncology, Polish Mother’s Memorial Hospital Research Institute, 281 Rzgowska Str., 93-338 Lodz, Poland; jan.bienkiewicz@umed.lodz.pl; 2 Institute of Medical Biology, Polish Academy of Sciences, 106 Lodowa Str., 93-232 Lodz, Poland; mkielbik@cbm.pan.pl (M.K.); mklink@cbm.pan.pl (M.K.); 3 Department of Molecular Medicine, Medical University of Lodz, Al. Kościuszki 4, 90-419 Lodz, Poland; daria.domanska@umed.lodz.pl; 4 Department of Clinical Pathology, Polish Mothers’ Memorial Hospital-Research Institute, 281 Rzgowska Str., 93-338 Lodz, Poland; tk.pracownia@gmail.com (T.K.); hanna-romanowicz@wp.pl (H.R.); 5 The Central Laboratory of Medical University in Lodz, 6/8 Mazowiecka Str., 92-215 Lodz, Poland; bozena.szymanska@umed.lodz.pl; 6 Gynecologic Oncology Center, Department of Obstetrics and Gynecology, First Faculty of Medicine, Charles University in Prague and General University Hospital in Prague, 128 00 Prague, Czech Republic; Filip.Fruehauf@vfn.cz; 7 Department of Surgical and Endoscopic Gynecology, Medical University in Lodz, Al. Kościuszki 4, 90-419 Lodz, Poland; amalinowski@kki.pl

**Keywords:** miRNA, ovarian cancer, survival, prognostic factor

## Abstract

**Simple Summary:**

MiRNA-103/107-DICER axis may be one of the key regulators of cancer aggressiveness. Data on miRNA-103/107 in high grade serous ovarian cancer is scarce. We aimed to assess miRNA-103/107 expression levels in high grade serous ovarian cancer tissues and relate them to patients’ clinicopathological data. MiRNA-103/107, DICER expression levels were also evaluated in selected ovarian cancer cell lines. Clinical and prognostic significance of miRNA-103/107 was not confirmed in our study. However, the results of our study support the possible existence of miRNA-103/107- DICER axis in ovarian cancer.

**Abstract:**

High levels of miRNA-103/107 are associated with poor outcomes in the case of breast cancer patients. MiRNA-103/107-DICER axis may be one of the key regulators of cancer aggressiveness. MiRNA-103/107 expression levels have never been related to patients’ clinicopathological data in epithelial ovarian cancer. We aimed to assess miRNA-103/107 expression levels in high grade serous ovarian cancer tissues. Expression levels of both miRNAs were related to the clinicopathological features and survival. We also evaluated expression levels of miRNA-103/107 and DICER in selected ovarian cancer cell lines (A2780, A2780cis, SK-OV-3, OVCAR3). We assessed the relative expression of miRNA-103/107 (quantitative reverse transcription-polymerase chain reaction) in fifty archival formalin-fixed paraffin-embedded tissue samples of primary high grade serous ovarian cancer. Then, miRNA-103/107 and DICER expression levels were evaluated in selected ovarian cancer cell lines. Additionally, DICER, N-/E-cadherin protein levels were assessed with the use of western blot. We identified miRNA-107 up-regulation in ovarian cancer in comparison to healthy tissues (*p* = 0.0005). In the case of miRNA-103, we did not observe statistically significant differences between cancerous and healthy tissues (*p* = 0.07). We did not find any correlations between miRNA-103/107 expression levels and clinicopathological features. Kaplan–Meier survival (disease-free and overall survival) analysis revealed that both miRNAs could not be considered as prognostic factors. SK-OV-3 cancer cell lines were characterized by high expression of miRNA-103/107, relatively low expression of DICER (western-blot), and relatively high N-cadherin levels in comparison to other ovarian cancer cell lines. Clinical and prognostic significance of miRNA-103/107 was not confirmed in our study.

## 1. Introduction

Ovarian cancer is characterized by a high fatality rate and is responsible for approximately 2–3% of all cancer deaths. The early-stage disease has a 5-year survival of 93%. Unfortunately, the majority of patients are diagnosed at the FIGO III or IV stage of the disease, for which the 5-year survival is much lower [1]. Epithelial ovarian cancer (EOC) is the most common type of ovarian malignancy, as only 10% of tumors are of non- epithelial origin. Serous high-grade carcinomas (HGSOC) are the most prevalent type of EOC, which are characterized by TP53 mutations and relatively poor outcome [2].

MicroRNAs (miRNAs) are small (approximately 18–25 nt), single-stranded non-coding RNAs that are evolutionarily conserved among species [3]. MiRNA genes are transcribed by RNA polymerase II to pri-miRNAs, which are cleaved by the microprocessor complex (nuclease Drosha together with DGCR8 protein) to create hairpin-like pre-miRNAs. Pre-miRNAs bind to the Exportin- 5 (RanGTP-dependent transporter) and are exported to the cytoplasm. RNase III-type nuclease enzyme DICER splits pre-miRNAs into two single-stranded forms of miRNA (miRNA-3p and miRNA-5p). One strand creates the mature miRNA, and the second passenger strand is usually destroyed. However, it is possible that the passenger strand can also be selected as a mature form of miRNA. Mature forms of miRNAs are incorporated into the RNA-induced silencing complex (RISC) and bind to the 3′ untranslated regions (UTRs) of their mRNA targets, which causes posttranscriptional suppression/activation of translation or mRNA cleavage [3,4]. MiRNAs possess unique abilities to affect the expression of genes and take part in such cellular processes as proliferation, differentiation, invasion, migration, epithelial-to-mesenchymal transition (EMT), or apoptosis [3,4]. Numerous authors showed miRNAs to be dysregulated in multiple cancers. Aberrant expression of miRNAs has been reported in multiple neoplasms and related to the stage of the disease or clinical outcome [5,6]. A large body of evidence suggests that miRNAs play a crucial role in carcinogenesis, tumor progression, and metastasis. Several miRNAs may be up-regulated in specific neoplasms; however, a global miRNA reduction in human cancers seems to be a common phenomenon [7]. A potential explanation for a global decrease in miRNA expression may be inhibition of DICER. Down-regulation of DICER has been detected in epithelial ovarian cancer (EOC). Meritt et al. reported that low DICER expression was associated with advanced-stage disease and reduced median survival [8]. Martello et al. identified a miRNA family (miRNA-103/107) that inhibited miRNA biosynthesis by targeting DICER [9]. They reported that high levels of miR-103/107 are associated with metastasis and poor outcome in breast cancer. Inhibition of miRNA-103/107 in malignant cells resulted in the attenuation of migratory and metastatic properties. Martello et al. concluded that the up-regulation of miRNA-103/107 is responsible for the induction of EMT, attained by down-regulating miRNA-200 levels. However, the role of miRNA-103/107 in EOC has not been elucidated yet, as the evidence is scarce. The Cancer Genome Atlas (TCGA) provided data on miRNA-103/107 expression levels in EOC [10]. Yang et al. identified miRNA-103 as an oncogene in serous ovarian cancer, which promotes invasion and metastasis via the down-regulation of DICER1 [11]. Apart from those two studies, there is no evidence on the clinical significance of both miRNAs. Several authors reported aberrant miRNA103/107 expression levels in other tumors. Yu et al. identified miRNA-103/107 up-regulation in bladder cancer specimens and revealed its oncogenic role in cell proliferation and PI3K/AKT signaling partially through PTEN dependent mechanism [12]. However, some authors showed that miRNA-107 might also be considered as a tumor suppressor. Tang et al. described that ectopic expression of miRNA-107 suppressed cell proliferation and was associated with the down-regulation of cyclin E1 (CCNE1) expression [13].

Although miRNA-103/107 may be one of the key-regulators of EOC carcinogenesis through the down-regulation of DICER, its prognostic and clinical significance has not been thoroughly evaluated. Thus, we decided to investigate miRNA-103/107 expression levels in primary HGSOC tissues and relate it to clinicopathological characteristics, with particular attention to overall survival (OS) and progression-free survival (PFS).

## 2. Results

### 2.1. miRNA-103/107 Evaluation in FFPE Tissues

We included fifty patients who were diagnosed with serous high-grade ovarian cancer between 2010 and 2018 (clinical data are presented in Table 1).

**Figure 1:**
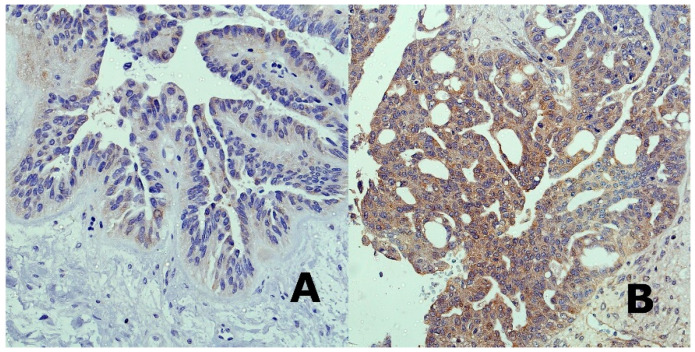
IHC (original magnification 40×) staining of ovarian cancer tissues: (**A**) Weak cytoplasmic positivity for DICER, (**B**) strong cytoplasmic positivity for DICER.

Quantitative Real-time PCR was performed in fifty formalin-fixed paraffin-embedded (FFPE) samples of high grade serous ovarian cancer and ten FFPE samples of normal Fallopian tubes’ fimbriae containing normal surface epithelium. Fallopian tubes’ fimbriae were chosen as controls, since the majority of serous carcinomas appear to arise from lesions in the distal fallopian tube [14]. An experienced pathologist assessed all samples. Careful microdissection of representative tissue areas was performed. MiRNA-103/107 expression was assessed in all fifty ovarian cancer samples and controls. We were not able to evaluate DICER expression, since mRNA derived from FFPE tissues is sensitive to chemical modifications and degradation. MiRNAs are known to be stable and less affected by the embedding process and degradation over time [15]. We performed immunohistochemical analysis of DICER in our set of FFPE tissue samples (Figure 1). We discovered that majority of ovarian cancer samples showed a reduced level of DICER protein. We found only six samples which were characterized by strong cytoplasmic positivity in cancer cells. What is worth mentioning is the fact that among six samples with strong positivity, there were five samples with low miRNA-103/107 levels.

**Figure 2:**
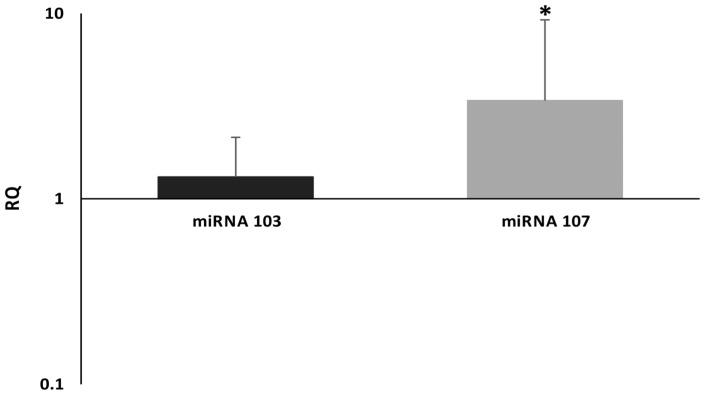
MiRNA-103 and -107 expression in ovarian cancer and control tissue samples. Statistically significant higher expression of miRNA-107: * Mann–Whitney U test, *p* = 0.0005. RQ = 1 for control tissue samples.

MiRNA-107 expression levels, which were determined in primary high-grade ovarian cancer tissues, showed up-regulation in comparison to Fallopian tubes’ fimbriae containing normal surface epithelium (Figure 2, mean RQ for cancer—1.69, lower quartile—0.99, upper quartile—2.99; mean RQ for controls—0.6, lower quartile—0.55, upper quartile—0.89; *p* = 0.0005). In the case of miRNA-103, we did not observe statistically significant differences between cancerous and healthy tissues. However, miRNA-103 showed a trend towards up-regulation in high-grade ovarian cancer samples (mean RQ for cancer—1.24, lower quartile—0.67, upper quartile—1.66; mean RQ for controls—0.77, lower quartile—0.63, upper quartile—0.94; *p* = 0.07). Furthermore, we found a positive correlation between miRNA-103 and miRNA-107 expression values (Spearman’s rank correlation coefficient, *p* = 0.00006). We evaluated miRNA-103/107 expression levels regarding such clinicopathological data as overall survival, progression-free survival, FIGO stages, platinum sensitivity, Ca125/HE4 levels, age, and BMI (body mass index). Statistical analysis revealed that there was no correlation between FIGO stages and expression levels of selected miRNAs (ANOVA, miRNA-103—*p* = 0.45; miRNA-107—*p* = 0.25). Similarly, we did not find any significant association between miRNA-103/107 expression levels and BMI (Spearman’s rank correlation coefficient, miRNA-103—*p* = 0.25; miRNA-107—*p* = 0.83) or age (Spearman’s rank correlation coefficient, miRNA-103—*p* = 0.19; miRNA-107—*p* = 0.75). Spearman’s rank correlation coefficient indicated that neither Ca125 nor HE4 showed any relationship with expression levels of selected miRNAs (Spearman’s rank correlation coefficient, miRNA-103: Ca125—*p* = 0.54/HE4—*p* = 0.35; miRNA-107: Ca125–*p* = 0.26/HE4—*p* = 0.17). We also evaluated miRNA-103/107 expression levels in the early stage versus the advanced stage of cancer, however, we did not find any correlations (FIGO stage I vs. II, III, IV, Mann–Whitney U test: miRNA-103—*p* = 0.8, miRNA-107—*p* = 0.3). Expression levels (low vs. high) of both miRNAs did not differ between platinum-sensitive and platinum-resistant patients (chi-square test, *p* = 0.11). MiRNA103/107 expression levels in regard to all clinicopathological data are presented in Table 2.

### 2.2. Survival Analysis

**Figure 3:**
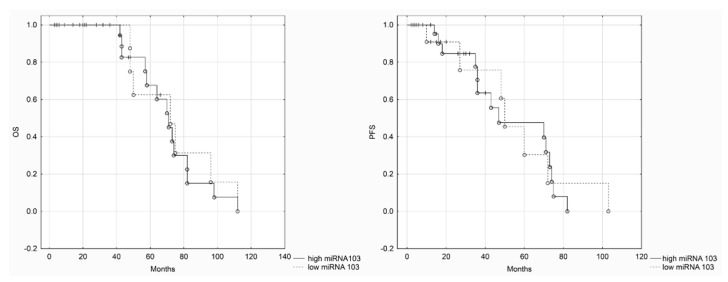
Kaplan–Meier analysis of OS (overall survival) and PFS (progression-free survival)—miRNA-103 (long rank test, *p* = 0.68, *p* = 0.78, respectively).

**Figure 4:**
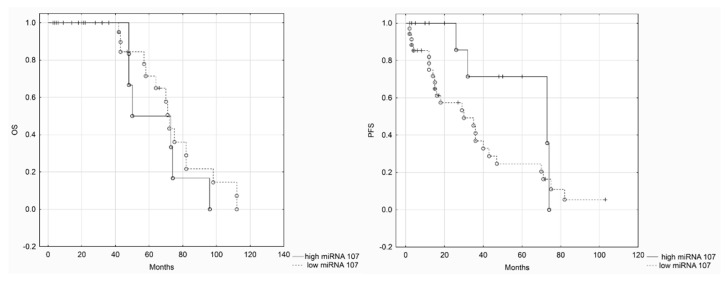
Kaplan–Meier analysis of OS (overall survival) and PFS (progression-free survival)—miRNA-107 (long rank test, *p* = 0.34, *p* = 0.09, respectively).

We divided patients into two groups based on miRNA expression levels (low and high miRNA-103/107) in order to perform Kaplan–Maier survival analysis. Both DFS (disease-free survival) and OS (overall survival) were assessed in regard to miRNA-103/107 expression levels (Figure 3 and Figure 4). Statistical analysis revealed that there were not any significant differences in survival between patients in low and high miRNA-103/107 subgroups.

**Figure 5:**
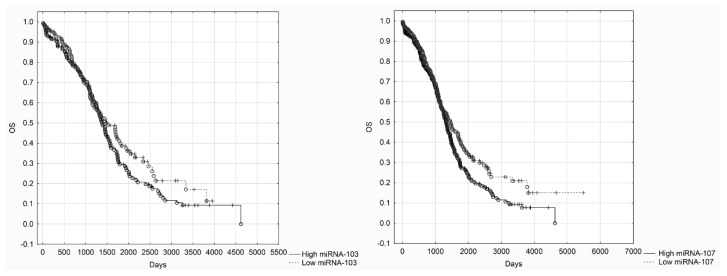
Kaplan–Meier analysis of OS (overall survival) in the Cancer Genome Atlas (TCGA) cohort—miRNA-103 and miRNA-107 (long rank test, *p* = 0.16, *p* = 0.01, respectively).

We also decided to perform survival analysis (OS) based on the data derived from TCGA. OncoLnc is a commonly available tool for exploring survival correlations, and for downloading clinical data coupled to expression data for miRNAs [16]. OncoLnc contains data from studies performed by TCGA. We used data from TCGA cohort of 470 ovarian cancer patients and performed Kaplan–Meier analysis. Due to the high number of cases, we decided to compare the bottom third to the top third according to expression values (cut-off values: RQ for the bottom third 43.8, RQ for the upper third 77.1). Kaplan–Meier survival analysis of TCGA data revealed that patients characterized by high miRNA-107 levels had more favorable overall survival. The survival difference between patients with low and high miRNA-107 levels was significant. However, we did not find any significant differences in survival between patients in low and high miRNA-103 subgroups (Figure 5).

### 2.3. Evaluation of miRNA-103/107, DICER, and N-/E-Cadherin in Selected Ovarian Cancer Cell Lines

After miRNA-103/107 evaluation in FFPE tissues, we also decided to assess miRNA-103/107 expression levels in the most commonly used, commercially available ovarian cancer cell lines. We hypothesized that potential up-regulation of miRNA-103/107 might be associated with lower DICER expression levels and partial loss of E-cadherin. Such findings would support the possible existence of the miRNA-103/107-DICER axis in ovarian cancer. According to Martello et al., the miRNA-103/107-DICER axis plays a crucial role in epithelial plasticity and fosters the invasive behavior of cancer cells [9]. Since we failed to show any significant clinical correlations in regard to miRNA-103/107 in high grade serous ovarian cancer, we decided to perform a preliminary and simple assessment of various cancer cell lines in order to verify the potential existence of the miRNA-103/107-DICER axis in the case of ovarian cancer. We decided to evaluate DICER, N-/E-cadherin, and miRNA-103/107 expression levels in selected ovarian cancer cell lines. Loss of E-cadherin, together with the presence of N-cadherin are hallmarks of epithelial to mesenchymal transition. We decided to choose cell lines that are commonly used as a template for ovarian cancer: A2780 (cisplatin-sensitive, EC50 = 6 µM) and A2780cis (cisplatin-resistant, EC50 = 30 µM) cell lines; group 2—resistant to cisplatin, but differing in the degree of aggressiveness SK-OV-3 (aggressive, EC50 = 38µM) and OVCAR-3 (non-aggressive, EC50 = 20 µM). SK-OV-3, OVCAR -3, A2780, and A2780cis cancer cell lines present different properties in regard to aggressiveness, chemo-sensitivity, epithelial character, or metastatic potential. SK-OV-3 is considered to be an aggressive and chemoresistant ovarian cancer cell line, probably derived from clear cell carcinoma. OVCAR-3 is a typical epithelial serous-like ovarian cancer cell line that seems to be modestly resistant to cisplatin and less aggressive than SK-OV-3. A2780 (cisplatin not resistant) and A2780cis (cisplatin-resistant) cancer cell lines are probably derived from undifferentiated or endometrioid ovarian carcinoma [17].

**Figure 6:**
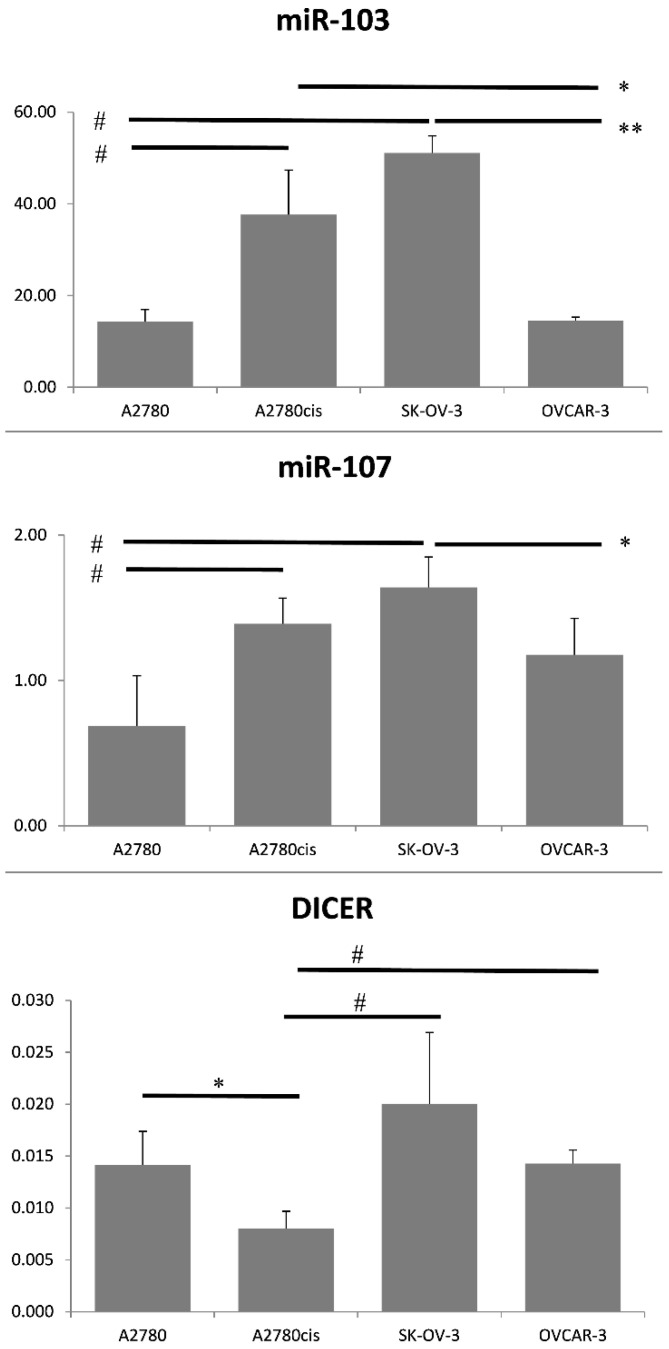
mRNA level of DICER, miRNA-103, and miRNA-107 in A2780, A2780cis, SK-OV-3, and OVCAR-3 cell lines. A2780, A2780cis, SK-OV-3, OVCAR3 ovarian cancer cell lines were cultured, harvested, and total RNA or miRNA was isolated from cells. DICER, miRNA-103, and miRNA-107 expression was measured using the qRT-PCR assay. Data are presented as means 2^−ΔCT^ ± SD from four independent experiments (*n* = 4). 2^−ΔCT^ is an absolute value representing the expression level of each gene in a particular cell line. Statistically significant increase of miRNA-103: #A2780 vs. A2780cis or SK-OV-3, *p* ≤ 0.02 (Mann–Whitney U test). Statistically significant decrease of miRNA-103: * A2780cis vs. OVCAR-3; ** SK-OV-3 vs. OVCAR-3, *p* ≤ 0.02 (Mann–Whitney U test). Statistically significant increase of miRNA-107: # A2780 vs. A2780cis or SK-OV-3, *p* ≤ 0.02 (Mann–Whitney U test). Statistically significant decrease of miRNA-107: * SK-OV-3 vs. OVCAR-3, *p* ≤ 0.02 (Mann–Whitney U test). Statistically significant decrease in the expression of DICER: * A2780 vs. A2780cis, *p* ≤ 0.007 (Mann–Whitney U test). Statistically significant increase in the expression of DICER: # A2780cis vs. SK-OV-3 or OVCAR-3, *p* ≤ 0.007 (Mann–Whitney U test).

**Figure 7:**
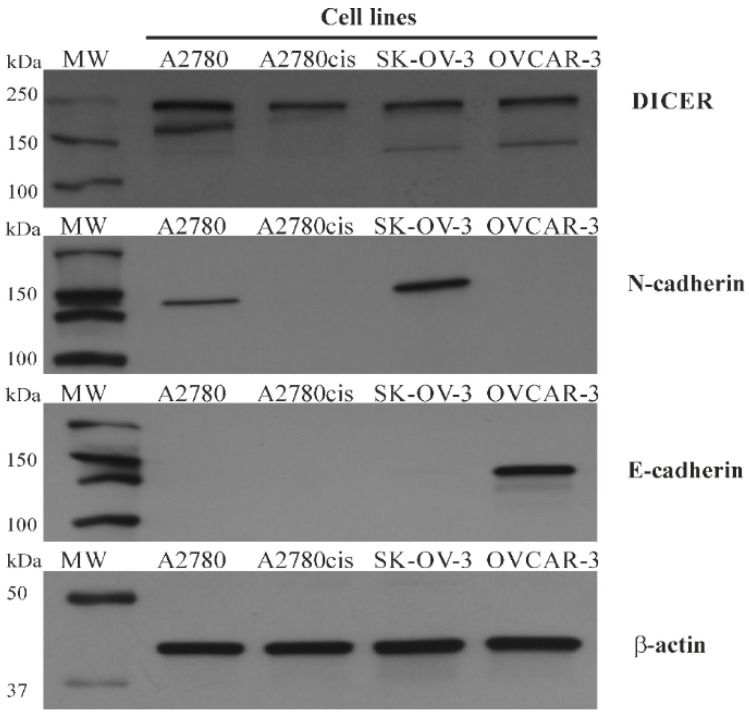
The total level of DICER, N-cadherin and E-cadherin proteins in A2780, A2780cis, SK-OV-3, and OVCAR-3 cell lines-Immunoblots. A2780, A2780cis, SK-OV-3, OVCAR3 ovarian cancer cell lines were cultured, harvested, and lysed with RIPA buffer. This Figure contains representative immunoblots of DICER, N-cadherin, E-cadherin, and β-actin proteins level, which were obtained with the Immunoblotting-ECL method.

**Figure 8:**
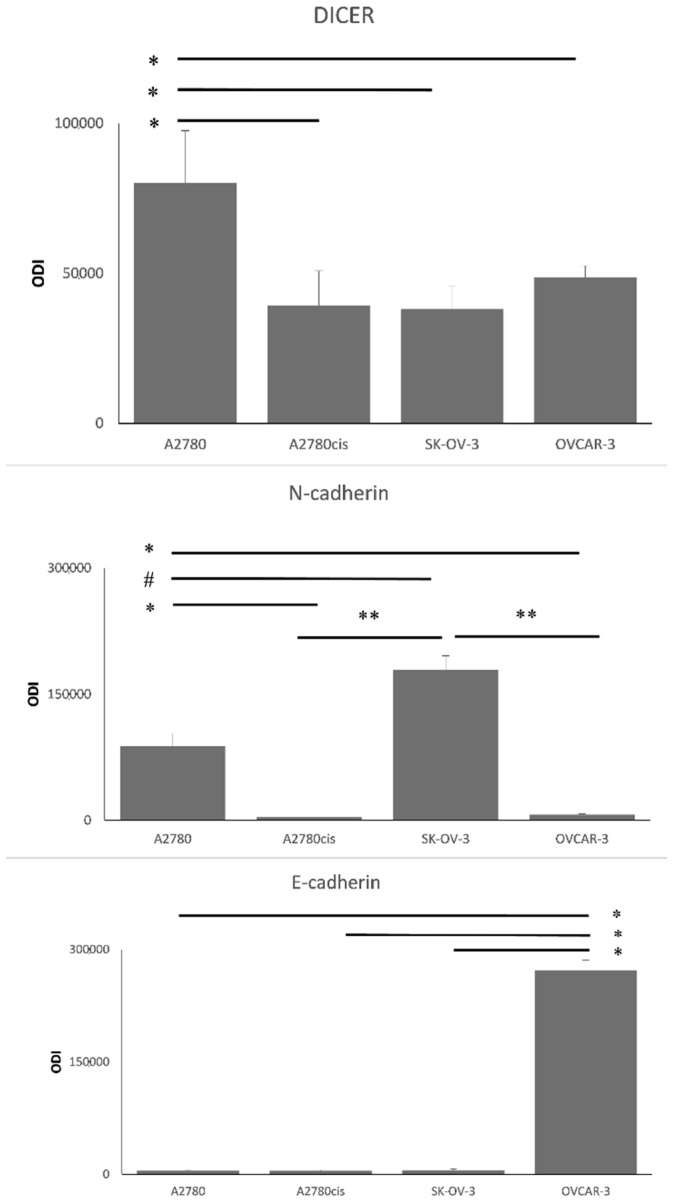
The total level of DICER, N-cadherin and E-cadherin proteins in A2780, A2780cis, SK-OV-3, and OVCAR-3 cell lines—densitometric analysis. A2780, A2780cis, SK-OV-3, OVCAR3 ovarian cancer cell lines were cultured, harvested, and lysed with RIPA buffer. The level of DICER, N-cadherin, E-cadherin, and β-actin proteins was evaluated with the Immunoblotting-ECL method. Bands were quantified by densitometric analysis, and data are presented as the optical density intensity (ODI) of the area under each band’s peak ± SD from five independent experiments (*n* = 5). Statistically significant decrease in the level of DICER: * A2780 vs. A2780cis or SK-OV-3 or OVCAR-3, *p* ≤ 0.007 (Mann–Whitney U test). Statistically significant decrease in the level of N-cadherin: * A2780 vs. A2780cis or OVCAR-3; ** SK-OV-3 vs. A2780cis or OVCAR-3, *p* ≤ 0.0006 (Mann–Whitney U test). Statistically significant increase in the level of N-cadherin: # A2780 vs. SK-OV-3, *p* ≤ 0.0006 (Mann–Whitney U test). Statistically significant decrease in the level of E-cadherin: * OVCAR-3 vs. A2780 or A2780cis or SK-OV-3, *p* ≤ 0.00005 (Mann–Whitney U test).

Such diversification among ovarian cancer cell lines allowed us to evaluate DICER, N-/E-cadherin, and miRNA-103/107 expression in molecularly different types of cells and increased our chances of detecting any correlations among them. We hypothesized that the cell lines with the highest miRNA-103/107 expression levels would be associated with low DICER, which would contribute to a partial loss of E-cadherin and higher expression of N-cadherin at the same time. The highest miRNA-103/107 expression levels were detected in SK-OV-3 and A2780cis ovarian cancer cells (Figure 6). We performed mRNA assessment by RT-PCR and did not detect low DICER expression in SK-OV-3 in comparison to other ovarian cancer cells (Figure 6). The discrepancy between mRNA levels and protein expression is a common phenomenon [18]. Thus, we decided to use the western blotting method in order to re-evaluate DICER expression in selected ovarian cancer tissues (Figure 7 and Figure 8).

Interestingly, there was a difference between the level of DICER protein assessed by western-blotting and the expression of its mRNA in the case of A2780 and SK-OV-3 cell lines. A low DICER protein level characterized SK-OV-3 cells as opposed to its mRNA level. A2780 cells were characterized by high DICER protein level as opposed to its mRNA level. We compared A2780 (cisplatin-sensitive) and A2780cis (cisplatin-resistant) ovarian cancer cells in regard to miRNA-103/107 and DICER expression levels. We found that level of DICER protein was significantly higher in A2780 cells in comparison to other tested cell lines (Mann–Whitney U test *p* ≤ 0.007). Furthermore, we observed higher expression levels of miRNA-103/107 in A2780cis cells (Figure 6; Mann–Whitney U test miRNA-103—*p* ≤ 0.02; miRNA-107—*p* ≤ 0.02). Similarly, SK-OV-3 (cisplatin-resistant) ovarian cancer cells showed significantly higher expression levels of miRNA-103/107 in comparison to A2780 cells (cisplatin-sensitive) (Figure 6; Mann–Whitney U test miRNA-103—*p* ≤ 0.02; miRNA-107—*p* ≤ 0.02).

## 3. Discussion

Our results showed that miRNA-107 is up-regulated in primary high-grade serous ovarian cancer tissues in comparison to normal epithelium derived from the Fallopian tube’s fimbriae. Nevertheless, we did not find any clinical correlations with miRNA-107 expression levels. We also did not confirm that miRNA-103 is up-regulated in cancerous tissues. Such results may be caused by a small number of samples (i.e., 50 patients). However, we believe that our investigation is not without merit. To the best of our knowledge, there have not been any studies on ovarian cancer that evaluated miRNA-103/107 expression levels in regard to clinicopathological data. The only data on miRNA-103/107 survival comes from TCGA research. We assessed the OS in TCGA cohort of patients. Kaplan–Meier analysis indicated that low miRNA-107 expression levels were associated with improved survival.

Expression levels of both miRNAs were also determined in other types of tumors, such as gastric or bladder cancers. MiRNA-103/107 expression was assessed by Yu et al. in bladder cancer samples. Both miRNAs were up-regulated in the bladder cancer specimens and positively correlated with the tumor stage [12]. Li et al. demonstrated that overexpression of miRNA-107 in gastric cancer might be associated with gastric cancer metastasis. Their results suggested that miRNA-107 promotes cancer metastasis through the down-regulation of DICER1 [19].

MiRNA-103/107- DICER axis has been described in breast cancer cells by Martello et al. According to them, invasive and metastatic properties of cancer cells are empowered by the up-regulation of miRNA-103/107 [9]. High levels of miRNA-103/107 affect DICER expression and cause its attenuation. Martello et al. associated the miRNA-103/107-DICER axis with epithelial to mesenchymal transition (EMT), identifying miRNA-200 family members as downstream mediators of the axis. Thus, high levels of both miRNAs may lead to more invasive and metastatic abilities of cancer cells. ZEB1 and ZEB2 are miRNA-200 targets and crucial genes for mesenchymality. According to Martello et al., both of them are down-regulated in antagomir-103/107-treated cells to about 50%. Additionally, the authors performed Kaplan–Meier survival analysis, which showed that high levels of miR-103/107 are associated with metastasis and poor outcome.

Identification of miRNAs that may control DICER expression levels is crucial in full understanding of miRNA-dependent carcinogenesis, tumor growth, and metastasis. Only a few authors have assessed MiRNA-103 expression levels in ovarian cancer. Wilczynski et al. assessed miRNA-103 by qRT-PCR in 48 samples derived from advanced serous ovarian cancer patients and found no differences between primary tumor and healthy ovarian tissue [20]. Yang et al. performed qRT-PCR to compare miR-103 expression levels in ovarian cancer and healthy ovarian tissues, however, they used only five fresh tissue samples of serous ovarian cancer [11]. Yang et al. showed that miRNA-103 was significantly up-regulated in ovarian cancer samples in comparison to healthy ovarian tissues. Furthermore, they reported that overexpression of miRNA-103 in cancer cell lines led to the enhancement of migration or invasion and a significant reduction of DICER1 levels.

DICER seems to be one of the key regulators of miRNA’s expression and action in cancerous cells. Several authors reported that DICER expression might be associated with patients’ prognosis. Meritt et al. performed qRT-PCR with validation by immunohistochemistry in 111 samples of EOC (2 endometrioid, 109 serous) and reported that low DICER expression was significantly associated with advanced tumor stage [9]. Moreover, they found that high DICER expression was associated with increased survival among EOC patients. Flavin et al. reported similar results and showed that high DICER expression might be associated with low metastatic lesions. However, survival analysis revealed that DICER expression did not affect the survival rates [21]. Such results stay in corcondance with our hypothesis. The results of survival analysis in TCGA cohort of patients showed that low miRNA-107 levels were associated with improved survival. The analysis of TCGA patients failed to show a possible impact of miRNA-103 levels of survival, but we believe it might have been changed if more patients had been included. Low miRNA-103/107 expression levels should be associated with high DICER levels. The up-regulation of DICER has been identified by Meritt et al. as a favorable factor in EOC patients. It seems that low levels of miRNA-107 and high levels of DICER should be both considered as a mark of improved prognosis among EOC patients.

Different DICER expression levels characterize ovarian cancer cell lines. Wang et al. reported low DICER levels in cisplatin-resistant A2780 cells in comparison to A2780 cisplatin-sensitive line. Furthermore, the down-regulation of DICER decreased the sensitivity of A2780 cancer cells and inhibited cisplatin-induced apoptosis [22]. In the study by Kuang et al., DICER down-regulation promoted cell proliferation and was significantly decreased in cisplatin-resistant A2780 cells compared with parental A2780 cells [23]. Our results showed that A2780cis is characterized by lower DICER expression levels than parental A2780 ovarian cancer cells. Moreover, we demonstrated that A2780cis cells presented a significantly higher expression of miRNA-103 and miRNA-107 values in comparison to A2780 cells. Such results indicate the possible existence of the miRNA-103/107-DICER axis in the case of ovarian cancer. High levels of both miRNAs were coexistent with DICER down-regulation in A2780cis cells. Furthermore, we found that SK-OV-3 cancer cells were characterized by the highest miRNA-103/107 expression levels and relatively low level of DICER protein among four selected ovarian cancer cell lines. It is worth noticing that SK-OV-3 cells also presented high levels of N-cadherin, which may be evidence for the possible shift of these cells towards the mesenchymal phenotype.

The possible existence of miRNA-103/107-DICER axis, that had previously been described in the case of breast cancer, convinced us to perform a study dedicated to the clinical usefulness of miRNA-103/107 expression levels in high-grade serous ovarian cancer patients. We did not aim to define the exact molecular ways of action of both miRNAs. MiRNA-103/107 expression levels have never been related to prognostic clinicopathological tumor characteristics. The results of the study indicated that miRNA-103/107 did not have any clinical or prognostic significance in our sample of patients. Furthermore, evaluation of DICER in FFPE tissues was not possible, due to the high amount of degraded mRNA. We found that majority of our serous high-grade ovarian cancer samples were characterized by reduced levels of DICER protein in immunohistochemistry, and it is what we expected in regard to the potential existence of the miRNA-103/107-DICER axis. Therefore, we aimed to perform a preliminary evaluation of SK-OV-3, OVCAR-3, A2780, and A2780cis cancer cell lines in order to verify a possible existence of the miRNA-103/107-DICER axis in the case of ovarian cancer. We selected various ovarian cancer cell lines that are derived from different histotypes of EOC. We expected that selected cells should vary between each other in regard to DICER and miRNA-103/103 expression levels. The cells with the highest miRNA-103/107 expression levels should be characterized by the lowest DICER expression at the same time. Our results confirmed that the miRNA-103/107-DICER axis might exist in the case of ovarian cancer, however, we are far from drawing any final conclusions.

Lack of clinical significance of both miRNAs might be related to the small set of patients or the fact that up-regulation of miRNA-103/107 may happen only in few ovarian cancer cells that gain migratory potential and form metastases.

## 4. Materials and Methods

Ethical approval was obtained from the Polish Mother’s Memorial Hospital Research Institute Ethics Committee (21 May 2019, approval number 71/2019). All individuals who participated in the study provided their consent. All procedures performed in studies involving human participants were in accordance with the ethical standards of the institutional and/or national research committee and with the 1964 Helsinki declaration and its later amendments or comparable ethical standards. All patients were operated in the Department of Operative Gynecology, Endoscopy and Gynecologic Oncology, Polish Mother’s Memorial Hospital Research Institute, Lodz, Poland. Total hysterectomy with bilateral salpingo-oophorectomy, omentectomy, appendectomy was performed in all cases. The extent of the surgery was individually modified in order to obtain optimal cytoreduction. Standard platinum-taxane chemotherapy was introduced in all cases as a first-line treatment. We defined platinum-resistant tumors when there was a relapse/progression within six months after completion of the chemotherapy. Serum levels of CA125 (cancer antigen 125), serum human epididymis antigen-4 (HE4), and ROMA index (Risk of Malignancy Algorithm) were obtained from patients.

### 4.1. Assessment of Formalin-Fixed, Paraffin-Embedded Tissues

RNA isolation was performed with the use of a miRNeasy FFPE Kit (Qiagen, Hilden, Germany), according to the manufacturer’s protocol. Spectrophotometry (PicoDrop, 260/280 nm) was used in order to assess the quality of the samples. Reverse transcription was performed with the use of the TaqMan^®^ MicroRNA Reverse Transcription Kit (Applied Biosystems, Waltham, MA, USA) and miRNA-specific primers (RT primer), according to the manufacturer’s protocol.

Quantitative Real-time PCR was performed with the use of standard TaqMan^®^ MicroRNA Assays (Applied Biosystems): hsa-miR-103 (Assay ID: 000439), hsa-miR-107 (Assay ID: 000443) and RNU6B (endogenous control, Assay ID: 001093). The 10 μL qPCR reaction mixture included 0.7 μL RT product, 5 μL TaqMan Fast PCR Master Mix (Applied Biosystems) and 1 μL TaqMan miRNA Assay (20×). The reactions were incubated in a 96-well plate at 95 °C for 10 min, followed by 40 cycles of 95 °C for 5 s and 60 °C for 20 s. All reactions were run in duplicate (Applied Biosystems 7900HT Fast Real-Time PCR System). We used Detection System 2.3 Software (Applied Biosystems, Waltham, MA, USA) for the quantification of miRNA. Relative expression was calculated according to the Ct method 2^−ΔΔCt^.

### 4.2. Quantitative Real-Time PCR Method for Selected Ovarian Cancer Cell Lines

A2780 and A2780cis were purchased from ECACC General Cell Collection (UK), while SK-OV-3, and OVCAR-3 were purchased from ATCC (USA). All cell lines are of epithelial origin, have adherent growth as a monolayer, and defined resistance or sensitivity to cisplatin. The growth medium consists of RPMI 1640 with 10% FBS and the addition of penicillin and streptomycin (100 U/mL/100 μg/mL). Moreover, cisplatin in the concentration of 1µM was added to the A2780cis cell line every 2–3 passages in order to maintain its resistance to cisplatin. A2780, A2780cis, SK-OV-3, OVCAR3 ovarian cancer cell lines were cultured on culture flasks until they reached 90% of confluence. All cell lines were harvested, centrifuged, and total RNA or miRNA was isolated from cells using TRIzol^®^ Reagent (Life Technologies, Waltham, MA, USA) or GeneMatrix Universal RNA/miRNA Purification Kit (EURX, Gdansk, Poland), respectively, according to the manufacturer procedure. Next, the quality control of isolated RNA and miRNA with the use of Nanodrop with ND 1000 Software (ThermoFisher Scientific, Waltham, MA, USA) was performed. RNA (5 μg) was processed directly to cDNA synthesis using Maxima First Strand cDNA Synthesis Kits for RT-qPCR (Life Technologies), according to the manufacturers manual. Similarly, the mature RNU6B, miR-103, and miR-107 were processed directly to cDNA with the use of the Taqman MicroRNA Reverse transcription kit. The human β-actin, DICER, RNU6B, miR-103, miR-107 expressions were quantified using TaqMan Gene Expression Assays (Applied Biosystems, CA, USA) by real-time PCR using ABI 7900-HT detection system (Applied Biosystems, Life Technologies), according to the manufacturer’s protocol. Controls with no template cDNA were performed with each assay. Relative quantitation of gene expression was calculated using the comparative CT (∆∆CT) method. The obtained data were analyzed with ABI 7900-HT (RQ manager software v1.2) and DataAssist software v3.01 and are presented as the RQ value- representing fold change in gene expression normalized to the reference genes (β-actin or RNU6B) and relative to the control. Additionally, data are presented as 2^−ΔCT^, which represents the absolute value of the mRNA level of each evaluated gene in a particular cell line.

### 4.3. Immunoblotting-ECL (Western Blotting Method)

A2780, A2780cis, SK-OV-3, OVCAR3 ovarian cancer cell lines were cultured on culture flasks until they reached 90% of confluence. All cell lines were harvested, centrifuged, and lysed with RIPA lysing buffer (ThermoFisher Scientific) with the addition of 1 mM PMSF and 1% of halt protease and phosphatase inhibition cocktail for 30 min on ice. The lysates were stored at −70°C until further analysis. The amount of protein in each sample was measured using a DC Protein Assay kit. The cell lysates containing equal levels of proteins were run on a 10% SDS-PAGE mini-protean precast TGX gel, and proteins were transferred to PVDF membranes using a Trans-Blot Turbo Transfer System (Bio-Rad, Hercules, CA, USA) at 2.5A for 10 min. Afterward, the membranes were blocked with SuperBlock Blocking Buffer for 30 min and blotted with mouse monoclonal anti-DICER (1:500, Thermo Fisher Scientific, catalog # MA5-31353,Waltham, MA, USA), rabbit monoclonal anti-N-cadherin (1:1000, Cell Signaling Technology, N-Cadherin (D4R1H) XP^®^ Rabbit mAb #13116), rabbit monoclonal anti-E-cadherin (1:1000, Cell Signaling Technology, E-Cadherin (24E10) Rabbit mAb #3195, Danvers, MA, USA) or mouse IgG anti-βactin antibody (1:4000, Thermo Fisher Scientific, catalog # MA1-744) (1 h, room temperature). After washing the membranes (5 times in 2× TBS-Tween 20), they were incubated with secondary antibodies, HRP-conjugated goat anti-rabbit IgG (1:4000) or HRP-conjugated goat anti-mouse IgG (1:4000) (1 h, room temperature), and again washed five times. Proteins were detected by the incubation of membranes with ECL Western Blotting Substrate. Proteins in blots underwent densitometric analysis using a FluoroChem MultiImage FC Cabinet (Alpha Innotech Corporation, San Leandro, CA, USA) and Alpha Ease FC software 3.1.2 (Alpha Innotech Corporation, San Leandro, CA, USA). The results are presented as the optical density intensity (ODI) of the area under each band’s peak.

### 4.4. Immunohistochemistry

Immunohistochemical staining was performed in formalin-fixed, paraffin-embedded tissues, which were cut into 4 μm slices on a microtome. Deparaffinization and rehydration were performed in xylene and ethanol (standard protocol). Slices were boiled in 0.01 M citrate buffer (pH 6.0) for 20 min, and then incubated with 3% hydrogen peroxide for 5 min. After washings with PBS, sections were incubated for 0.5 h with mouse DICER monoclonal antibody (1:400, Thermo Fisher Scientific, CL0378, Catalog # MA5-31353). After washes, a horseradish peroxidase kit was used for antibody detection and diaminobenzidine for chromagen visualization (Dako EnVision Detection Systems).

### 4.5. Statistical Analysis

Statistical analysis was performed with Excel 2013 (Microsoft, Redmond, Washington) and STATISTICA 13.1 software (Statsoft, Tulsa, OK, USA). Statistical significance was defined by *p* value lower than 0.05. Kruskal–Wallis test, median test (Pearson’s chi-squared test), unpaired t-test, and Mann–Whitney U-test were used. Relative expression levels (RQ values) were determined with the use of the delta-delta CT method, adjusted to the expression of selected endogenous control.

## 5. Conclusions

Further investigation may clarify the role of the miRNA-103/107-DICER axis in ovarian cancer and its potential clinical/prognostic significance. The results of our study show that miRNA-103/107 did not have any clinical or prognostic significance. Nevertheless, we believe that our study is not without merit, as the evidence on the miRNA-103/107-DICER axis in ovarian cancer is extremely scarce.

## Figures and Tables

**Table 1:** Pathological characteristics and survival data.

**Serous, High-Grade Ovarian Cancer Patients**	
Cases number (N)	50
Age (range, years)	60.1 (30–83)
**FIGO Stage**	
I	6
II	1
III(N)	30
IV(N)	13
**Recurrence/Progression**	
No (N)	9
Yes (N)	41
**Platinum-Resistant**	
No (N)	38
Yes (N)	12
PFS (Range, months)	29 (2–103)
OS (Range, months)	42.3 (14–112)
**Overall Survival**	
No (N)	28
Yes (N)	22

PFS—progression-free survival (until the first recurrence/progression); OS—overall survival.

**Table 2:** Basic results of miRNA-103/107 expression regarding clinicopathological data.

	MiRNA-103	MiRNA-107
Mean RQ	1.69	1.24
miRNA expression vs. control tissue, *p* values	A trend towards up-regulation, *p* = 0.07	Up-regulation, *p* = 0.0005
Clinicopathological data, *p* values		
BMI	*p* = 0.25	*p* = 0.84
Age	*p* = 0.19	*p* = 0.75
FIGO stage	*p* = 0.45	*p* = 0.25
FIGO stage I vs. II,III,IV	*p* = 0.8	*p* = 0.3
Ca125	*p* = 0.54	*p* = 0.26
HE4	*p* = 0.35	*p* = 0.17
Deceased vs. Alive	*p* = 0.46	*p* = 0.39
Progression (yes vs. no)	*p* = 0.45	*p* = 0.06
Platinum-resistance (yes vs. no)	*p* = 0.37	*p* = 0.21

## References

[1] Howlader N., Noone A.M., Krapcho M., Miller D., Brest A., Yu M., Ruhl J., Tatalovich Z., Mariotto A., Lewis D.R. (2018). SEER Cancer Statistics Review, 1975–2016.

[2] Vang R., Shih I.-M., Kurman R.J. (2009). Ovarian Low-grade and High-grade Serous Carcinoma: Pathogenesis, Clinicopathologic and Molecular Biologic Features, and Diagnostic Problems. Adv. Anat. Pathol..

[3] Bartel B. (2009). MicroRNAs: Target Recognition and Regulatory Functions. Cell.

[4] Ambros V. (2004). The functions of animal microRNAs. Nature.

[5] Ventura A., Jacks T. (2009). MicroRNAs and Cancer: Short RNAs Go a Long Way. Cell.

[6] Chen C.-Z. (2005). MicroRNAs as oncogenes and tumor suppressors. N. Engl. J. Med..

[7] Kumar M.S., Lu J., Mercer K.L., Golub T.R., Jacks T. (2007). Impaired microRNA processing enhances cellular transformation and tumorigenesis. Nat. Genet..

[8] Merritt W.M., Lin Y.G., Han L.Y., Kamat A.A., Spannuth W.A., Schmandt R., Urbauer D., Pennacchio L.A., Cheng J.-F., Nick A.M. (2008). Dicer, Drosha, and Outcomes in Patients with Ovarian Cancer. N. Engl. J. Med..

[9] Martello G., Rosato A., Ferrari F., Manfrin A., Cordenonsi M., Dupont S., Enzo E., Guzzardo V., Rondina M., Spruce T. (2010). A MicroRNA Targeting Dicer for Metastasis Control. Cell.

[10] TCGA Research Network (2011). Integrated genomic analyses of ovarian carcinoma. Nature.

[11] Yang Z.Y., Wei X., Zhou X.S., Liu Y., Gong C., Gao Q.L. (2014). MicroRNA-103 promote ovarian cancer metastasis through targeting Dicer1. Chin. J. Cancer Prev. Treat..

[12] Yu Q.F., Liu P., Li Z.Y., Zhang C.F., Chen S.Q., Li Z.H., Zhang G.Y., Li J.C. (2018). MiR-103/107 induces tumorigenicity in bladder cancer cell by suppressing PTEN. Eur. Rev. Med. Pharmacol. Sci..

[13] Tang Z., Fang Y., Du R. (2019). MicroRNA-107 induces cell cycle arrests by directly targeting cyclin E1 in ovarian cancer. Biochem. Biophys. Res. Commun..

[14] Erickson B.K., Conner M.G., Landen C. (2013). The role of the fallopian tube in the origin of ovarian cancer. Am. J. Obstet. Gynecol..

[15] Liu A., Xu X. (2011). MicroRNA isolation from formalin-fixed, paraffin-embedded tissues. Breast Cancer.

[16] Anaya J. (2016). OncoLnc: Linking TCGA survival data to mRNAs, miRNAs, and lncRNAs. PeerJ Comput. Sci..

[17] Shaw T.J., Senterman M.K., Dawson K., Crane C.A., Vanderhyden B.C. (2004). Characterization of intraperitoneal, orthotopic, and metastatic xenograft models of human ovarian cancer. Mol. Ther..

[18] Vogel C., Marcotte E. (2012). Insights into the regulation of protein abundance from proteomic and transcriptomic analyses. Nat. Rev. Genet..

[19] Li X., Zhang Y., Shi Y., Dong G., Liang J., Han Y., Wang X., Zhao Q., Ding J., Wu K. (2011). MicroRNA-107, an oncogene microRNA that regulates tumour invasion and metastasis by targeting DICER1 in gastric cancer. J. Cell. Mol. Med..

[20] Wilczynski M., Żytko E., Danielska J., Szymańska B., Dzieniecka M., Nowak M., Malinowski J., Owczarek D., Wilczyński J.R. (2018). Clinical significance of miRNA-21, -103, -129, -150 in serous ovarian cancer. Arch. Gynecol. Obstet..

[21] Flavin R., Smyth P.C., Finn S.P., Laios A., O’Toole S.A., Barrett C., Ring M., Denning K.M., Li J., Aherne S.T. (2008). Altered eIF6 and Dicer expression is associated with clinicopathological features in ovarian serous carcinoma patients. Mod. Pathol..

[22] Wang X., Chen H., Wen Y., Yang X., Han Q., Jiang P., Huang Z., Cai J., Wang Z. (2018). Dicer affects cisplatin-mediated apoptosis in epithelial ovarian cancer cells. Mol. Med. Rep..

[23] Kuang Y., Cai J., Li D., Han Q., Cao J., Wang Z. (2013). Repression of Dicer is associated with invasive phenotype and chemoresistance in ovarian cancer. Oncol. Lett..

